# Supplementary material for: Analyzes In Silico Indicate the lncRNAs MIR31HG and LINC00939 as Possible Epigenetic Inhibitors of the Osteogenic Differentiation in PDLCs
Source: Genes (Basel). 2023 Aug 18;14(8):1649. doi: 10.3390/genes14081649 (PMC10454380; doi:10.3390/genes14081649)
Supplement: Supplementary file 1 [file genes-14-01649-s001.zip › genes-2534656-supplementary.docx]

**Table S1.** Gene expression primers sequences and polymerase chain reaction cycle conditions.

| **Gene** | **Primers** | **5’ - 3’ sequence** | **Reaction Conditions** | **Product Size (pb)** |
| --- | --- | --- | --- | --- |
| ***MIR31HG* (ENST00000304425.3)** | Forward | GGGTGATTGAGGGCTCTACA | 95 °C, 10 s  56 °C, 15 s  72 °C, 20 s | 180 |
|  | Reverse | AATGACTGGTCTACGTGGGG |  |  |
|  |  |  |  |  |
| ***LINC00939-201***  **(ENST00000502479.1)** | Forward | TCCCATGCACTGTCTCCATT | 95 °C, 10 s  56 °C, 15 s  72 °C, 20 s | 192 |
|  | Reverse | GGGAGCCAAAGAAATGCCAA |  |  |
|  |  |  |  |  |
| ***SP7***  **(ENST00000536324.4)** | Forward | ATGAGTGGGAAAAGGGAGGG | 95 °C, 10 s  60 °C, 15 s  72 °C, 20 s | 190 |
|  | Reverse | TCAACAACTCTGGGCAAAGC |  |  |
|  |  |  |  |  |
| ***DLX4***  **(ENST00000240306.5)** | Forward | AGGCTCTGCTCCATTCCTTT | 95 °C, 10 s  57 °C, 15 s  72 °C, 20 s | 178 |
|  | Reverse | GCTTCGCCTCAGATGATGTG |  |  |

**Figure S1 – Search results for poly(A) sites on the genomic regions of the *MIR31HG* and *LINC00939* lncRNAs:** The blues and red columns represent the poly(A) site clusters, identified in the PolyASite database, for the plus and minus strands, respectively, on the genomic regions of the *MIR31HG* (**A**) and *LINC00939* (**B**) lncRNAs. The dotted red lines highlighted the poly(A) site clusters in 3’UTR region.


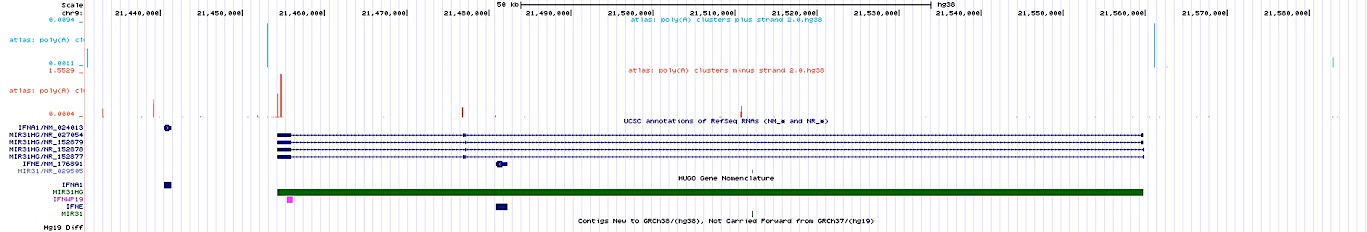

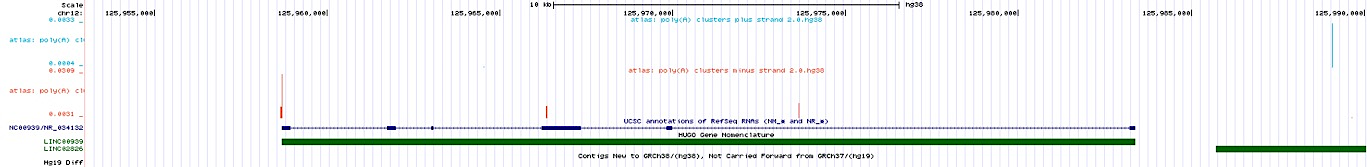


**A**

**B**
